# Innervation of papillary thyroid cancer and its association with extra-thyroidal invasion

**DOI:** 10.1038/s41598-020-58425-5

**Published:** 2020-01-30

**Authors:** Christopher W. Rowe, Tony Dill, Nathan Griffin, Phil Jobling, Sam Faulkner, Jonathan W. Paul, Simon King, Roger Smith, Hubert Hondermarck

**Affiliations:** 1 School of Medicine and Public Health, University of Newcastle, Callaghan, NSW 2308 Australia; 2 Department of Endocrinology, John Hunter Hospital, Locked Bag 1, Newcastle, NSW 2310 Australia; 3 Hunter Medical Research Institute, 1 Kookaburra Circuit, New Lambton Heights, 2305 NSW Australia; 4 Department of Anatomical Pathology, NSW Health Pathology (Hunter), Locked Bag 1, HMRC, Newcastle, NSW 2310 Australia; 5 School of Biomedical Sciences and Pharmacy, University of Newcastle, Callaghan, NSW 2308 Australia; 6 Present Address: ACT Pathology, Canberra Health Services, ACT Government, Canberra Hospital, Canberra, ACT Australia

**Keywords:** Thyroid cancer, Cancer microenvironment

## Abstract

Nerves are emerging regulators of cancer progression and in several malignancies innervation of the tumour microenvironment is associated with tumour aggressiveness. However, the innervation of thyroid cancer is unclear. Here, we investigated the presence of nerves in thyroid cancers and the potential associations with clinicopathological parameters. Nerves were detected by immunohistochemistry using the pan-neuronal marker PGP9.5 in whole-slide sections of papillary thyroid cancer (PTC) (n = 75), compared to follicular thyroid cancer (FTC) (n = 13), and benign thyroid tissues (n = 26). Nerves were detected in most normal thyroid tissues and thyroid cancers, but nerve density was increased in PTC (12 nerves/cm^2^ [IQR 7–21]) compared to benign thyroid (6 nerves/cm^2^ [IQR: 3–10]) (p = 0.001). In contrast, no increase in nerve density was observed in FTC. In multivariate analysis, nerve density correlated positively with extrathyroidal invasion (p < 0.001), and inversely with tumour size (p < 0.001). The majority of nerves were adrenergic, although cholinergic and peptidergic innervation was detected. Perineural invasion was present in 35% of PTC, and was independently associated with extrathyroidal invasion (p = 0.008). This is the first report of infiltration of nerves into the tumour microenvironment of thyroid cancer and its association with tumour aggressiveness. The role of nerves in thyroid cancer pathogenesis should be further investigated.

## Introduction

Emerging data show that many solid tumours are innervated and that nerves actively participate in cancer progression^1^. Tumour denervation in prostate^2,3^, gastric^4,5^ and pancreatic cancers^6,7^ reduces tumour growth and invasion; and the presence of nerves is associated with metastases and increased tumour grade. The stimulatory role of nerves appears to be related to the release of neurotransmitters (such as norepinephrine) by nerve endings in the tumour microenvironment, resulting in the activation of neurosignalling in both stromal and cancer cells and the promotion of tumour progression. For instance, adrenergic neurosignalling induced by sympathetic nerves stimulates an angio-metabolic switch in endothelial cells, while cholinergic neurosignalling has been shown to activate cancer stem cell growth^4,5^. The growth of nerves in cancer is stimulated by the release of neurotrophic growth factors, such as nerve growth factor (NGF) from cancer cells^5,6,8–10^, resulting in an increased nerve density in the tumour microenvironment. Together, nerves, as well as neurotrophic growth factors are increasingly regarded as potential biomarkers and therapeutic targets in cancer.

In thyroid cancer, the presence and potential significance of nerves in the tumour microenvironment has not been investigated. Although it is known that the normal thyroid follicular epithelium is infiltrated by adrenergic, cholinergic and peptidergic nerves^11–14^, with nerve signalling causing measurable physiological effects on the secretion of thyroid hormone^15,16^ and on thyroid growth^17^, the presence and significance of nerves in thyroid cancer is unclear. Perineural invasion has rarely been described and appears to occur in only 2% of thyroid tumours^18^ but this may simply reflect the rarity of nerves in the thyroid cancer microenvironment.

In this study, we aimed to clarify the presence and quantity of nerves in the microenvironment of thyroid cancer. Nerves were detected in a cohort of thyroid cancers and benign thyroid tissues, and the association between innervation and clinicopathological parameters was investigated. The density of nerves was found to be increased in papillary thyroid cancer (PTC) compared to follicular thyroid cancers (FTC) and benign thyroid tissues. Interestingly, nerve density was positively associated with extra-thyroidal invasion.

## Materials and Methods

### Thyroid specimens

This study was prospectively approved by the Hunter New England Human Research Ethics Committee, who granted a waiver of consent for access to archival pathology material and approved the experimental protocol (HNE HREC 16/04/20/5.13). All study methodologies were carried out in accordance with relevant guidelines and regulations. Surgical resection specimens were processed and stored according to standard clinical and pathological procedures, then archived as formalin-fixed, paraffin-embedded blocks at a tertiary hospital pathology department (NSW Health Pathology, John Hunter Hospital, Lookout Road, New Lambton Heights, Australia). Archived blocks containing sections of lobes from thyroid cancers and benign thyroid pathology were obtained by electronic database search at a ratio of 4 cases of thyroid cancer for 1 benign control. 114 whole blocks of thyroid lobe specimens from 102 patients were included, comprising 26 cases of benign pathology, and 88 cases of thyroid cancer (PTC n = 75, FTC n = 13). 12 cases with malignant pathology in the ipsilateral lobe also had the contralateral lobe included in analysis due to separate pathology in the contralateral lobe (PTC [n = 4], follicular adenoma [n = 5] or multinodular change [n = 3]. No lobe was included twice. All histological samples were reviewed by an independent pathologist to confirm or revise the original diagnosis. Relevant clinical parameters were extracted from medical records.

Demographic and clinical characteristics of the included cases are presented in Table 1. Overall, patients with benign and malignant thyroid diseases were of a similar age (53.6 vs 56.4 years, p = 0.50), gender (70% vs 66% female, p = 0.78) and baseline thyroid function (TSH 0.95 vs 1.8 mIU/L p = 0.21).

**Table 1:** Demographic and clinical characteristics of thyroid samples.

	Benign thyroid	Thyroid Cancer	p
n	26	88	
Age (years)	56.4 ± 15.7	53.3 ± 18.6	0.45
Female gender (n, %)	18 (70%)	59 (67%)	0.83
TSH (mIU/L)*	0.95 ± 1.3	1.8 ± 1.2	0.94
**Pathology (n, %)**			
Papillary cancer		75 (85%)	
Follicular cancer		13 (16%)	
Normal thyroid	1 (4%)		
Multinodular	16 (62%)		
Follicular adenoma	9 (34%)		
**Tumour characteristics**			
Tumour size (cm)		2.5 ± 1.9	
Multifocality (n, %)		29 (33%)	
Extra-thyroidal invasion (n, %)		49 (56%)	
Microscopic		38/49	
Macroscopic		11/49	
Nodal metastases (n, %)		58 (66%)	

^*^TSH data missing for 15 cases with thyroid cancer, and 2 cases with benign pathology.

### Immunohistochemistry

Whole-slide sections (4 µm) were prepared from formalin-fixed paraffin-embedded blocks of benign and malignant thyroid tissue. Immunohistochemistry (IHC) for the pan-neuronal marker protein gene-product 9.5 (PGP9.5)^19^ was performed using the Ventana Discovery automated slide stainer (Roche Medical Systems, Tuscon, Az). The primary antibody was anti-rabbit polyclonal PGP9.5 antibody (catalogue number #Ab15503, Abcam, Cambridge, United Kingdom) at 1:600 dilution; previously validated in our laboratory^10,20^ and re-optimized for the automated platform. Negative controls were prepared using non-specific IgG isotype controls, and without addition of primary antibody (Supplementary Fig. S1), while positive controls were included on each slide. Antigen-retrieval was performed using Ribo-CC solution, (pH 6, Ventana, Roche), with secondary anti-rabbit HQ for 16 min at 37 °C (Ventana, Roche), and tertiary anti-HQ (Ventana, Roche) for 16 min at 37 °C. Manual counterstaining was performed using Mayer’s hematoxylin for 10 sec and Scott’s Tap Water for 30 sec, followed by dehydration, clearing and mounting. Staining of primary tumours for the precursor for nerve growth factor (proNGF) was performed using the automated Ventana platform and the anti-rabbit polycloncal proNGF antibody (Ab9040, Merck Millipore, Darmstadt, Germany) at a dilution of 1:350.

Subtyping of nerves was performed on a subgroup of thyroid cancers (n = 5) using antibodies against tyrosine hydroxylase (TH) for adrenergic (sympathetic) nerves (catalogue number #ab152, Abcam, Cambridge United Kingdom, 1:250 dilution); vesicular acetylcholine transporter (VAChT) for cholinergic (parasympathetic) nerves (catalogue number #ab62140, Abcam, 1:250 dilution) and anti-Substance P (SP) for peptidergic (sensory) nerves (catalogue number #ab14184, Abcam, 1:1000 dilution)^21^. Sections of innervated dermis and colonic mucosa were included as positive controls.

### Digital quantification of nerve density

**Figure 1:**
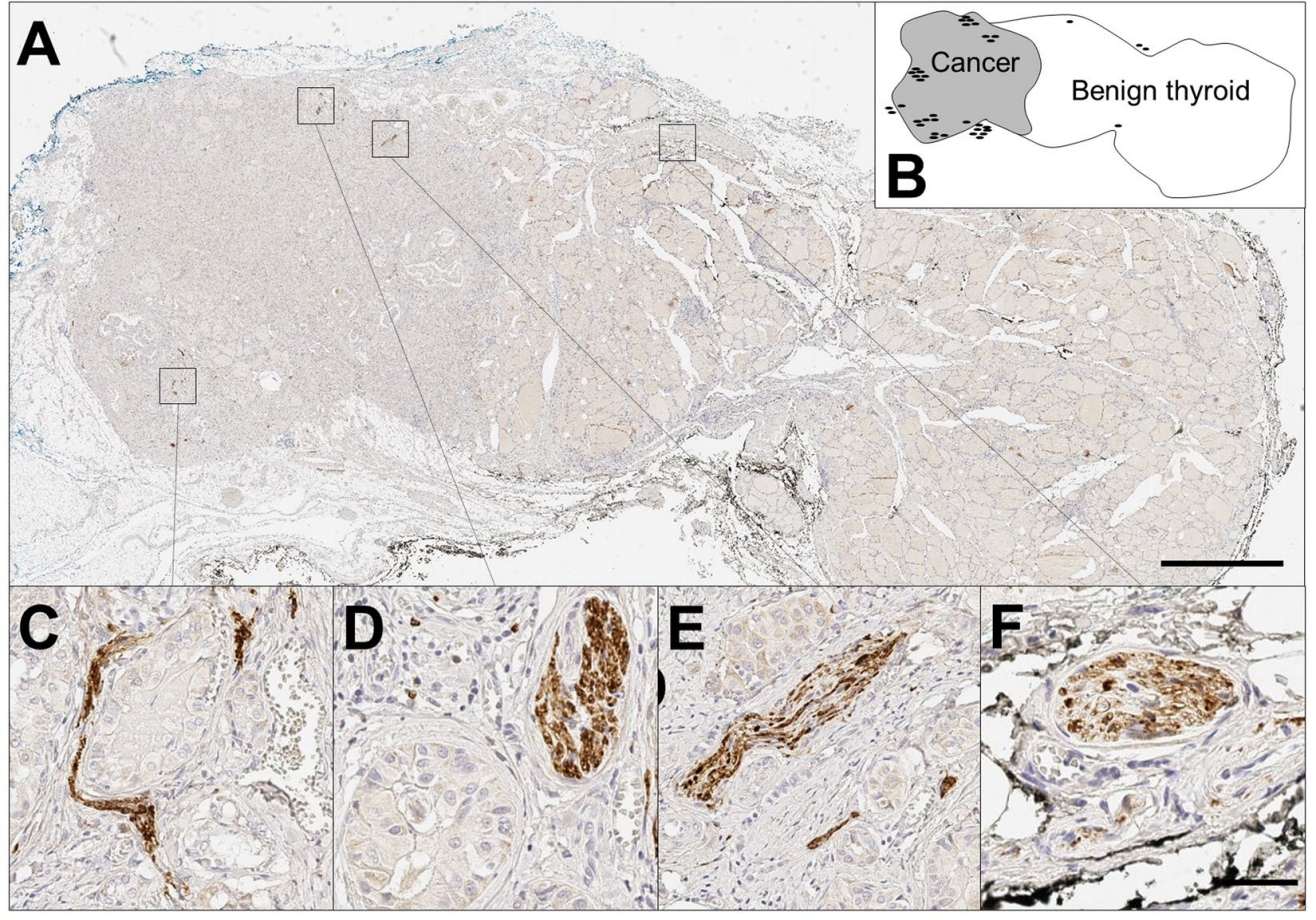
Nerves in benign and malignant thyroid tissues. **(A)** Low magnification (0.5×) of papillary thyroid cancer (left) and adjacent benign thyroid (right), stained using immunohistochemistry for PGP9.5 (pan-neuronal marker) and counterstained with haematoxylin. Scale bar 2 mm. **(B)** Schematic of A, showing location of benign and malignant tissue and nerves. Each black oval represents a single nerve trunk in approximate location. In this particular section there were 38 nerves (of which 34 were in the cancer, and 4 within benign thyroid), and the section contains 1 cm^2^ of thyroid tissue (including 0.32 cm^2^ of cancer). This gives a density of 38 nerves per cm^2^ of thyroid tissue; 108 cancer-associated nerves per cm^2^ of cancer; and 6 nerves per cm^2^ adjacent benign tissue. **(C–F)** High magnification (20×), demonstrating nerve trunks (brown stain), surrounded by papillary thyroid cancer cells **(C–E)**; or adjacent to the vascular bundle on edge of benign thyroid **(F)**. Scale bar 50 µm.

Prior to analysis, digitization of whole slides at 20× magnification was performed using the Aperio AT2 scanner (Leica Biosystems, Victoria, Australia). Tissue was analyzed using QuPath (Queens University, Belfast)^22^. Nerves were counted if they met the following criteria: immunoreactivity (positive PGP9.5 staining), typical anatomical appearance, and 3 or more axons visualized (for example, see Fig. 1C–F). Nerves with fewer than 3 axons were discounted as they could not reliably be distinguished from non-specific staining. Each discrete nerve segment was counted separately. Nerve density is presented as nerves per cm^2^ of thyroid tissue according to three parameters: (A) the number of thyroid-associated nerves (nerves within 1 mm of both benign and malignant thyroid tissue on a slide) divided by the total area of thyroid tissue present on the slide; (B) the number of cancer-associated nerves (nerves within 1 mm of intrathyroidal malignant cells) divided by the area of cancer present on the slide; and (C) the number of adjacent-benign associated nerves (thyroid-associated nerves present on a slide containing cancer, but >1 mm from malignant cells) divided by the area of benign tissue adjacent to the cancer on the slide. Separation distances of 1 mm were chosen to reflect a plausible maximum biological distance for paracrine signaling. In addition, perineural invasion (defined as tumour cells circumferentially surrounding a nerve^23^) was quantified for each case. Investigators were blinded to clinical and pathological characteristics of the included cases during slide analysis.

QuPath was further used to determine the intensity of cytoplasmic proNGF staining in cancer cells (quantified by h-score, an index between 0–300, calculated as the sum of 3× % of pixels with strong staining +2× % of pixels with intermediate staining +1× % pixels with weak staining).

### Statistical analysis

Group levels means and standard deviations (SD) are presented for parametric data, assessed with unpaired Student’s t-tests; with medians and interquartile ranges (IQR) presented for non-parametric data, compared using Wilcoxon RankSum (unmatched pairs) or SignRank (matched pairs) tests. Distribution was assessed using histograms and q-q plots. Analysis of association between nerve density and clinical and pathological parameters was undertaken by fitting separate log-linear regression models with log-transformed nerve-density as the dependent variable, and including model variables of age, sex, tumour size, extra-thyroidal invasion, multifocality and the presence of lymph node metastases. Separate models were fitted for each nerve density measure. Categorical data was assessed using Pearson’s Chi-square, with logistic regression models used to assess impact of potential confounding. Significance was assessed using a two-sided alpha of 0.05. All analyses were performed using Stata (version 14.2, Statacorp, College Station, Texas, USA).

## Results

### Nerve detection in thyroid cancer vs normal thyroid tissue

Using the pan-neuronal marker PGP9.5, nerves were detected in sections of PTC and FTC as well as benign thyroid tissues. Nerves were identified in 23/26 (88%) of exclusively-benign sections and 86/88 (98%) of sections containing malignant tissue, with nerves typically found in the periphery of the tumours (Fig. 1A,B) or invading deep inside the tumour (Fig. 1C–E), sometimes co-located in the neuro-vascular bundle (Fig. 1F).

### Nerve density is increased in PTC compared to FTC and benign thyroid tissue

Nerve density was quantified and found to be higher in sections containing thyroid cancer (median 10.3 nerves per cm^2^, IQR 5.9–20.2) than in sections containing exclusively-benign thyroid tissue (6.6 nerves per cm^2^, IQR 2.8–9.7, p = 0.005, Table 2A). When stratified by cancer subtype, the increase in nerve density was found to be associated exclusively with PTC: 12.4 [6.6–21.4] nerves per cm^2^ on sections containing PTC vs 6.6 [2.8–9.7] nerves per cm^2^ on sections containing exclusively-benign thyroid, p = 0.0008. For FTC, no increase in nerve density was seen (Table 2A). To determine whether there was evidence of clustering of nerves around the cancer, a subset of 62 sections containing both benign and malignant tissue on the same section were analyzed (Table 2B). In PTC, nerve density was significantly higher in the tumour (17.8 [8.0–41.2] nerves per cm^2^) than the adjacent benign regions (9.6 [5.7–18.0] nerves per cm^2^, p = 0.0008]). However, nerve density in the benign tissue adjacent to PTC was also higher than nerve density in exclusively-benign thyroid tissue (9.6 [5.7–18.0] vs 6.6 [2.8–9.7] nerves per cm^2^, p = 0.03).

**Table 2:** Nerve density in benign and malignant thyroid tissue.

	Measure	n	Area of Tissue (cm^2^)	Nerve Density (cm^2^)	P value*
A	Thyroid section containing cancer	88	2.0 (1.5–2.6)	10.3 (5.9–20.2)	0.005*
	• Thyroid section containing PTC	75	1.9 (1.5–2.6)	12.4 (6.6–21.4)	0.0008*
	• Thyroid section containing FTC	13	2.6 (2.3–2.9)	3.4 (2.5–4.5)	ns*
	Exclusively-benign thyroid section	26	1.9 (1.6–2.3)	6.6 (2.8–9.7)	Reference
B	**Region analysis (PTC only)**				
	• PTC slide-region only	62	0.6 (0.2–1.2)	17.8 (8.0–41.2)	0.0008^#^
	• Benign thyroid adjacent to PTC	62	0.7 (0.3–1.3)	9.6 [5.7–18.0]	0.027*

Data are median (IQR). Wilcoxon Ranksum or SignRank test used. *Comparator is ‘Exclusively-benign thyroid section’. ^#^Comparator is ‘Benign thyroid adjacent to PTC’. n = number of cases. ns = not significant.

### Resection technique does not affect assessment of nerve density

Analysis was performed on a subgroup of exclusively-benign sections (n = 19) and PTC sections (n = 13) resected under identical surgical conditions of total thyroidectomy and without lymph node dissection to determine whether surgical methodology (hemithyroidectomy vs total thyroidectomy) or resection extent (presence or absence of simultaneous lymph node dissection) may be affecting the assessment of nerve density presented in Table 2. The pattern of increased nerve density in PTC compared to benign thyroid was confirmed (17.2 vs 6.6 nerves per cm^2^ thyroid tissue respectively, p = 0.002), providing corroboration that the increase in nerve density observed around PTC is independent of the resection technique used (Supplementary Table S2). Further, using the full PTC and benign tissue dataset we constructed a log-linear regression model using nerve density per cm^2^ thyroid tissue as the dependent variable, and including model variables of presence of cancer, type of thyroidectomy, and nodal clearance as binary variables. In the base model, the presence of cancer remained highly associated with nerve density (p = 0.002), and there was no material change in parameter estimates or significance (p = 0.007) when including model variables of operation type and nodal clearance (Supplementary Table S3).

### The majority of nerves in PTC are of adrenergic nature

**Figure 2:**
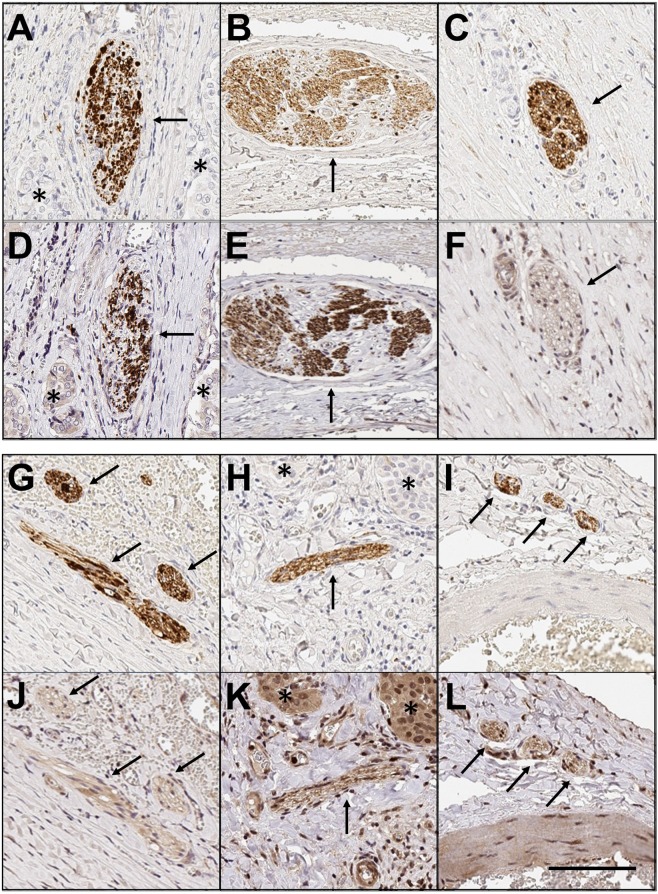
Subtyping of autonomic nerves. Paired images demonstrating serial sections through nerves in thyroid cancer, where the first image **(A–C,G–I)** shows a nerve immunolabelled with PGP9.5, and the second image **(D–F,J–L)** shows the same nerve labelled with a second neural immunostain. Pairs **(A,D)** and **(B,E)** show strong co-staining with PGP9.5 and Tyrosine Hydroylase (adrenergic neuronal marker). Pairs **(C,F)** and **(G,J)** show co-staining between PGP9.5 and Vesicular acetylcholine transporter (VACHT, cholinergic neuronal marker). Pairs **(H,K)** and **(I,L)** show co-staining for PGP9.5 and Substance P (peptidergic neuronal marker). Note also the staining of some malignant cells with Substance P in panel (K). 10× magnification. Black arrows indicate labelled nerves. Stars indicate malignant cells (where present). Scale bar: 100 µm.

Using the pan-neuronal marker PGP9.5 labelling as a reference, nerve subtypes were identified using neuronal markers specific for adrenergic, cholinergic and peptidergic nerves in serial sections. Serial sections were immunostained for either TH, VAChT or SP, to identify adrenergic, cholinergic and peptidergic nerves respectively. Strong, specific co-staining with PGP9.5 and TH was observed in the majority of nerves (for example, see paired images in Fig. 2A,D and B,E), demonstrating a high proportion of adrenergic nerves in the tumour microenvironment. To quantify the proportion of adrenergic nerves, 4 medium-power fields (10× magnification) with PGP9.5 positive nerves were identified for each case (n = 5). Corresponding nerve labelling was counted for matched fields, yielding the percentage of sympathetic nerve staining. Using this method, 55/69 nerves (80%) showed strong concordant immunoreactivity for PGP9.5 and TH. Immunostaining for parasympathetic nerves (VAChT, paired images in Fig. 2C,F and G,J) and peptidergic nerves (Fig. 2H,K and I,L) was present, but markedly less compared to sympathetic staining and limited to a subset of axons within the nerves, rendering a precise quantification of nerve density difficult. In SP stained sections, co-staining of the adjacent cancer cells was also noted (Fig. 2K), which has been previously reported in gastric cancer^24^.

### Nerve density and perineural invasion in PTC are positively associated with extra-thyroidal invasion

Multiple log-linear regression models were used to assess the relationship between nerves and clinical parameters of PTC, using nerve density as the dependent variable, and including model variables of age, gender, tumour size (cm), presence of extra-thyroidal invasion (either microscopic or gross), presence of multifocality and presence of nodal metastases (Table 3). In both models, the presence of extra-thyroidal invasion had significant positive association with nerve density (p < 0.001). In contrast, tumour size was inversely associated with nerve density (p < 0.005). No association with multifocality or nodal metastases was noted, and there was no evidence of confounding from age or gender. To confirm that the association between increased nerve density and extrathyroidal extension was not being caused by large tumours with gross extra-thyroidal extension resulting in encasement of nerves in the interstitial space, we repeated the regression model after excluding cases of PTC with gross extra-thyroidal extension (n = 8) (Supplementary Table S4). The association between nerves and extra-thyroidal invasion remained highly significant, with a 211% increase (95% CI 129 to 346%, p = 0.004) in nerve density in tumours with microscopic extra-thyroidal invasion, providing supportive evidence that the increase in nerve density is not an artefact of tumour growth.

**Table 3:** Association between nerve density in PTCs and clinical/pathological parameters.

Model variable	Nerves per cm^2^ of thyroid tissue		Nerves per cm^2^ of PTC	
	Beta coefficient	*p-value*	Beta coefficient	*p-value*
Age	0 (0 to 0)	*0.27*	0 (0 to 0)	*0.77*
Sex	−0.2 (−0.7 to 0.3)	*0.33*	−0.2 (−0.7 to 0.3)	*0.42*
Tumour size (cm)	−0.3 (−0.5 to 0) *−27% (−44 to −10%)*	*0.003*	−0.5 (−0.8 to −0.4) *−55% (−74 to −36%)*	<*0.0001*
Extra-thyroidal invasion	0.8 (0.3 to 1.3) *123% (41 to 250%)*	*0.001*	1.1 (0.6 to 1.6) *209% (87 to 411%)*	<*0.0001*
Multifocality	0 (−0.4 to 0.5)	*0.84*	0 (0 to 0.2)	*0.96*
Nodal metastases	−0.1 (−0.6 to 0.4)	*0.78*	−0.3 (−0.8 to 0.3)	*0.34*
Intercept	3.3 (2.4 to 4.1)		3.9 (2.9 to 4.8)	

Multiple log-linear regression of log-transformed nerve density and untransformed markers of cancer aggressiveness in PTCs. Coefficients (95% CI) are reported. Percentage change of the untransformed variable is co-reported (italics) for significant model variables, where each 1 unit increase in the model variable is associated with a percentage change in nerve density.

We further explored the potential relationship between nerves and extra-thyroidal invasion in PTC by assessing perineural invasion, as either a continuous or dichotomized (present vs absent) variable. A total of 26/75 (35%) of PTC had evidence of perineural invasion, with a median 8 (IQR 3–13) involved nerves per section. On dichotomized univariate analysis, tumours with extra-thyroidal invasion were more likely to have evidence of perineural invasion (46%) than PTC without extra-thyroidal extension (22%, p = 0.03). There was no association between perineural invasion and tumour size, multifocality or nodal metastases. This relationship was then explored in a multiple logistic regression model, with presence of tumoural extra-thyroidal invasion as the dichotomized dependent variable, and including potential model variables of number of nerves with perineural invasion, age, gender, tumour size (cm), multifocality and presence of nodal metastases. In this model, the identification of each nerve with perineural invasion increased the odds of the tumour having extra-thyroidal invasion by 20% (odds ratio 1.2, 95%CI 1.05–1.38, p = 0.008). There was no evidence of confounding factors from other model variables. In this model, tumour size (OR 1.08, 95% CI 1.02–1.14, p = 0.01) was also associated with extrathyroidal invasion. The presence of tumour multifocality failed to reach a p-value < 0.05 (OR 3.09, 95%CI 0.93–10.2, p = 0.06).

### Nerve density in PTC may be associated with proNGF expression

**Figure 3:**
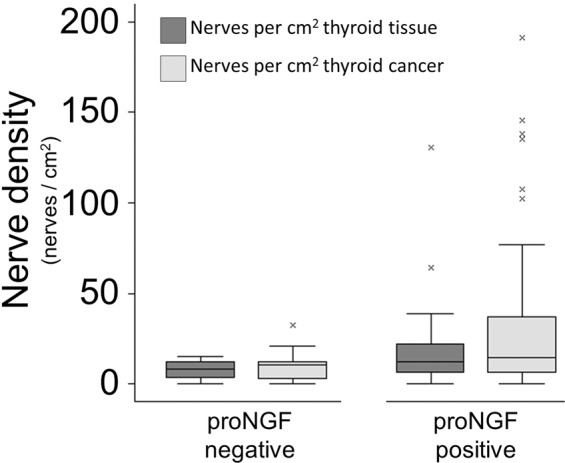
Nerve density in thyroid cancers, stratified by tumoural proNGF expression. Box (IQR) and whisker (5–95%) plot of nerve density, stratified by the presence or absence of proNGF expression in the primary tumour. Dark grey boxes compare density of nerves per cm^2^ of thyroid lobe containing PTC or FTC (medians 8.5 vs 11.4, p = 0.10). Light grey boxes compare nerve density per cm^2^ of PTC or FTC (medians 10.3 vs 14.3 p = 0.07).

In light of previous data showing proNGF expression in thyroid cancers^21^, and given that in prostate cancer proNGF has been reported as a driver of nerve infiltration^9^, we examined whether there was an association between proNGF expression in primary thyroid tumours and nerve density. ProNGF expression was quantified in primary tumours (n = 83) using immunohistochemistry and h-score (staining intensity). Dichotomizing positive proNGF expression at a h-score of 25, 86% (71/83) of thyroid cancers expressed proNGF. Median proNGF h-score was 55 (IQR 33–78) for all tumours, and was not significantly different between papillary (56, IQR 39–78) and follicular (39, IQR 25–78, p = 0.30) subtypes. Tumours expressing proNGF showed a greater density of nerves than tumours with negative proNGF expression (Fig. 3), although statistical significance was limited (p = 0.07). There were a median 14.3 nerves per cm^2^ thyroid cancer (IQR 6.0–36.8) associated with tumours expressing proNGF, compared to 10.3 (IQR 2.4–12.3) nerves per cm^2^ associated with cancers without proNGF expression (p = 0.07).

## Discussion

This paper clarifies the presence of nerves in thyroid cancer and demonstrates for the first time that nerve density is significantly higher in PTC compared to FTC and benign thyroid tissue. This relationship was robust, persisting when controlling for tumour size and other variables in multivariate models. Additionally, we have shown that these nerves were predominantly of adrenergic origin, although cholinergic and peptidergic nerves could also be occasionally detected; this is the first evidence for adrenergic nerves in PTC.

Tumour innervation has been established in several other malignancies^25^. For example, adrenergic innervation is demonstrated in breast cancer, where nerves infiltrate the tumour microenvironment and are associated with lymph node invasion^10,26^. In prostate cancer, both adrenergic and cholinergic nerves are present, and nerve density is increased inside and around prostate cancer compared to benign prostate, and correlates with more aggressive tumours^2,27,28^. Prostate cancer cells can drive neuronal growth (axonogenesis) *in vitro*^27^ and this neurotrophic effect is mediated through the release of proNGF^9,27^. In gastric cancer, cholinergic innervation is necessary for tumourigenesis and is driven by NGF release from cancer cells^4,5^. Increased nerve densities have also been found in the tumour microenvironment of colorectal^29^ and pancreatic^30^ carcinomas. Our data show a robust positive association between nerves in PTC and extra-thyroidal invasion. Both the density of nerves, and the presence of perineural invasion, were associated with extra-thyroidal invasion in independent analyses. The finding that perineural invasion is associated with aggressive tumours is consistent with data from other cancers^23^. Nerve density could therefore be used as a potential indication of PTC aggressiveness, and may assist with post-operative risk stratification of patients. However, further large scale clinicopathological investigations are warranted to confirm this hypothesis.

A further finding of our study is that nerve density in PTC may be associated with the expression of the neurotrophic factor proNGF by cancer cells, suggesting that proNGF could participate in the outgrowth of nerves in the tumour microenvironment and around the tumour. The role of proNGF/NGF in stimulating nerve outgrowth in the tumour microenvironment has already been described in prostate^9^, gastric^5^ and pancreatic^6^ cancers, where a NGF-adrenergic nerve feed-forward loop promotes nerve infiltration. Interestingly, the tyrosine kinase receptor TrkA, whose ligands include both NGF and proNGF, is expressed in nerves that have infiltrated the tumour microenvironment of thyroid cancer^20^, suggesting the hypothesis that proNGF/NGF could stimulate nerve outgrowth via signalling through TrkA receptors in nerve terminals infiltrated in thyroid cancer.

The predominance of adrenergic nerves PTC suggests the possibility of adrenoceptor-mediated signalling, either as an angiogenic switch on endothelium, as seen in prostate cancer^3^, and/or via direct effects on cancer cells, including cancer stem cells, as described in pancreatic cancer^6,7^. Functional alpha- and beta- adrenoceptors are present in benign thyroid tissue^14^, and adrenergic signalling is known to increase thyroid hormone production and mediate thyroid growth^15,31–33^. Beta-adrenoceptors have been demonstrated in thyroid cancer, where they are over-expressed compared to benign thyroid^34,35^. Therefore, similar to prostate and pancreatic cancers, the increased density of adrenergic nerves that we have detected in thyroid cancer could contribute to its progression and dissemination. The inverse association that we have observed between tumour size and nerve density also suggests that innervation may be important in the initiation of tumourigenesis, as described in gastric cancer^4^. Together, now that presence of nerves in thyroid cancer is clarified and given the association with extrathyroidal extension, further *in vitro* and *in vivo* animal experiments are warranted to explore the functional impact of nerves in thyroid cancer.

In conclusion, the demonstration that nerves are increased in PTC and correlate with extra-thyroidal invasion points to the potential clinical value of using nerve involvement as a biomarker of tumour aggressiveness in pathological analysis. In addition, these results suggest that similarly to prostate, gastric and pancreatic cancers, nerves may have a role in PTC progression.

## Supplementary Information

Supplementary Information.

## Data Availability

All data generated or analysed during this study are included in this published article (and its Supplementary Information files).
