# Supplementary material for: Innervation of papillary thyroid cancer and its association with extra-thyroidal invasion
Source: Sci Rep. 2020 Jan 30;10:1539. doi: 10.1038/s41598-020-58425-5 (PMC6992619; doi:10.1038/s41598-020-58425-5)
Supplement: Supplementary file 1 — Supplementary Information. [file 41598_2020_58425_MOESM1_ESM.pdf]

Supplementary Information for:

Innervation of papillary thyroid cancer and its association with extra-thyroidal invasion.

Christopher W Rowe[1,2,3], Tony Dill[4*], Nathan Griffin[3,5], Phil Jobling[5], Sam Faulkner[3,5], Jonathan W Paul[1,3], Simon King[4], Roger Smith[1,2,3] and Hubert Hondermarck[3,5]

Supplementary Figure S1

Negative controls for anti-PGP9.5 antibody (Abcam, catalogue number Ab15503). Immunostaining was performed without addition of primary antibody (shown). Scale bar: 100µm. Negative controls with substitution of the secondary antibody for non-specific IgG isotype controls were similar (not show).

(A): Section of human pancreas, 10× magnification. Islets of Langerhans are known positive controls for PGP9.5. Without addition of primary antibody, there is no staining of Islets, and no non-specific staining. Inset: Positive staining of Islet of Langerhans following addition of primary antibody.

(B): Human thyroid, 10× magnification. Without addition of primary antibody, there is no non-specific staining of thyroid follicular epithelium. Inset (A) shows normal thyroid following addition of correct primary antibody, demonstrating expected negative staining of thyroid follicular epithelium, allowing for straightforward identification of labelled nerves (See Figure 1 and 2). Inset (B) shows papillary thyroid cancer following addition of primary antibody, again showing absence of non-specific staining.

(A)

(B)

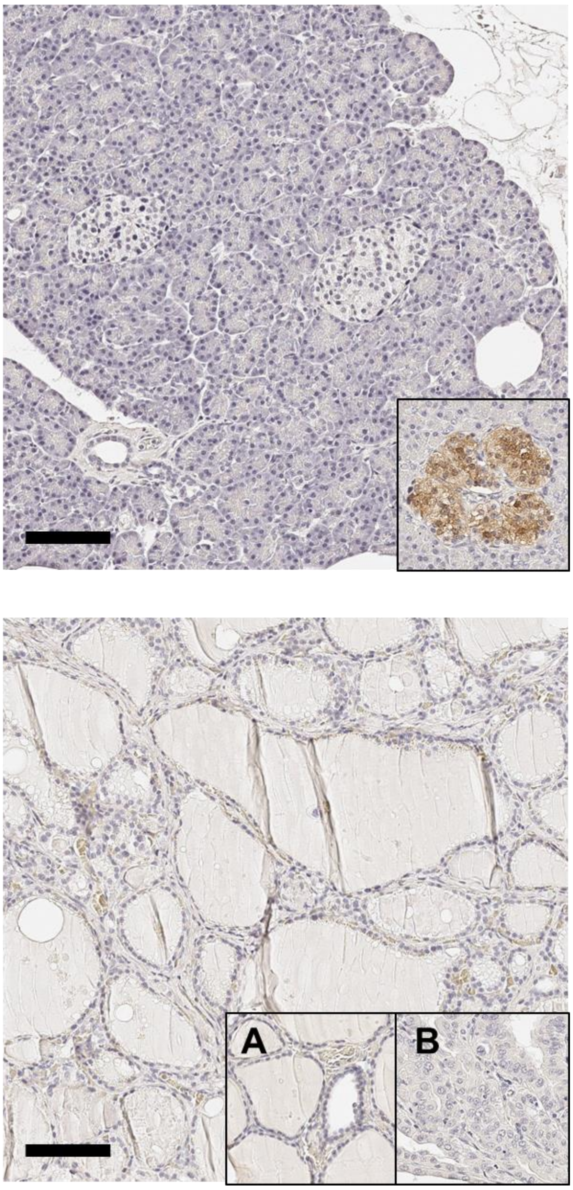

Supplementary Table S2: Subgroup analysis of nerve density around papillary thyroid cancers and benign thyroid tissue, resected under identical surgical conditions of total thyroidectomy and without lymph node dissection.

	**Benign thyroid**	**Papillary cancer**	*p-value*
Number of sections	19	13	
Age	54.3 ± 15.5	49.4 ± 20.9	*0.44*
Female gender (n, %)	15 (78%)	9 (69%)	*0.53*
TSH (mIU/L)^	0.73 ± 0.91	0.84 ± 0.62	*0.64*
Benign pathology			
Nodular goitre	11		
Follicular adenoma	8		
Total number of nerves	*11 (3 - 21)*	*23 (15 - 28)*	*0.048*
Total area of thyroid tissue (cm^2)	1.8 (1.7 – 2.3)	1.4 (0.9 – 1.9)	*0.10*
Nerves per cm^2 of thyroid tissue	*6.6 (2.9 – 9.8)*	*17.2 (11.2 – 18.8)*	*0.002*

Data are median (IQR). *Mann-Whitney test, using benign thyroid tissue as comparator. ^ TSH data missing for 2 benign cases and 1 malignant case.

Supplementary Table S3: Multiple log-linear regression of nerve density (dependent variable), including model variables of presence of cancer, type of operation (hemithyroidectomy = 0; total thyroidectomy = 1), and lymph node dissection (performed = 1) on the full cohort of benign thyroid tissue and papillary thyroid cancer. Inclusion of additional model variables around operation type did not change the overall parameter estimates or significance of the association between nerve density and thyroid cancer

**Base model**	**Beta coefficient (95% CI)**	*p-value*
Cancer present	0.69 (0.27 to 1.11)	0.002
**Exploratory model**		
Cancer present	0.67 (0.18 to 1.14)	0.007
Total thyroidectomy	0.50 (0.01 to 0.99)	0.05
Lymph node dissection	0.09 (-0.34 to 0.51)	0.69

Supplementary Table S4: Association between nerve density in PTCs and clinical/pathological parameters after excluding cases with gross extra-thyroidal invasion (n=8 with PTC and gross ETE excluded)

	**Nerves per cm^2 of thyroid tissue**		**Nerves per cm^2 of PTC**	
**Model variable**	Beta coefficient	*p-value*	Beta coefficient	*p-value*
Age	0 (0 to 0)	*0.45*	0 (0 to 0)	*0.86*
Sex	-0.3 (-0.8 to 0.2)	*0.26*	-0.2 (-0.8 to 0.3)	*0.42*
Tumor size (cm)	-0.3 (-0.5 to 0)<br>*-25% (-49% to 0%)*	*0.05*	-0.6 (-0.9 to 0.0)<br>*-62% (-89% to -34%)*	*<0.0001*
Extra-thyroidal invasion	0.75 (0.3 to 1.2)<br>*211% (129% to 346%)*	*0.004*	1.0 (0.4 to 1.5)<br>*261% (149% to 458%)*	*0.001*
Multifocality	0.1 (-0.4 to 0.5)	*0.75*	0 (-0.4 to 0.6)	*0.70*
Nodal metastases	0 (-0.5 to 0.5)	*0.96*	-0.2 (-0.8 to 0.4)	*0.48*
Intercept	3.1 (2.1 to 4.0)		3.9 (2.8 to 5.0)	

Multiple log-linear regression of log-transformed nerve density and untransformed markers of cancer aggressiveness in PTCs. Coefficients (95% CI) are reported. Percentage change of the untransformed variable is co-reported (italics) for significant model variables, where each 1 unit increase in the model variable is associated with a percentage change in nerve density.
